# Investigation of the effects of nicotinamide mononucleotide on kidney damage in rats with cecal ligation and perforation sepsis model

**DOI:** 10.1590/acb414926

**Published:** 2026-08-07

**Authors:** Ayla Arslan, Dilek Vural Keleş, Seçil Nazife Parlak, Seda Yakut, Habibe Gündoğdu, İrem Arslantürk, Gökçe Bağcı Uzun, Berna Öztürk Karagöz

**Affiliations:** 1 Ağri İbrahim Çeçen University – Faculty of Medicine – Department of Anatomy – Ağri – Turkey.; 2 Kirklareli University – Faculty of Health Sciences – Department of Nursing – Kirklareli – Turkey.; 3 Ağri İbrahim Çeçen University – Faculty of Medicine – Department of Histology and Embryology – Ağri – Turkey.; 4 Burdur Mehmet Akif Ersoy University – Faculty of Veterinary Medicine – Department of Histology and Embryology – Burdur – Turkey.; 5 Atatürk University – Faculty of Medicine – Department of Histology and Embryology – Erzurum – Turkey.; 6 Ağri İbrahim Çeçen University – Faculty of Medicine – Department of Medical Biochemistry – Ağri – Turkey.; 7 Malatya Turgut Özal University – Faculty of Medicine – Department of Anatomy – Malatya – Turkey.; 8 Ağri İbrahim Çeçen University – Faculty of Medicine – Department of Medical Pharmacology – Ağri – Turkey.

**Keywords:** Sepsis, Nicotinamide Mononucleotide, Kidney

## Abstract

**Purpose:**

To evaluate the protective effects of nicotinamide mononucleotide (NMN), a NAD+ precursor, on kidney tissue in rats with sepsis induced by cecal ligation and puncture model.

**Methods:**

In this study, 28 female Sprague Dawley rats were randomly divided into four equal groups: control, sham, sepsis, and sepsis+NMN (500 mg/kg, i.p.). Kidney tissues taken 24 hours after the sepsis model were histopathologically evaluated under light microscopy for tubular damage, glomerular congestion, and inflammatory infiltration parameters.

**Results:**

Histopathological examination revealed severe tubular injury, marked glomerular congestion, and dense inflammatory cell infiltration in the kidney tissues of the sepsis group. In the group treated with NMN, a significant reduction in these pathological findings was observed, while the glomerular structure and tubular architecture were preserved. Semi-quantitative histopathological scoring confirmed significantly increased renal injury in the sepsis group and significant attenuation of tissue damage following NMN treatment.

**Conclusion:**

The data obtained showed that NMN may play an important cytoprotective role in sepsis-induced acute kidney injury by preserving tissue architecture. More comprehensive molecular and biochemical studies are needed to fully elucidate the therapeutic potential and mechanisms of action of NMN.

## Introduction

More than 19 million people worldwide are affected by sepsis each year, which is a type of systemic inflammatory disorder that is associated with very high mortality rates^1^. Sepsis can occur because of various types of insults (*e.g.*, burns, severe trauma, hypotension/shock, peri-operational complications). The end result is that sepsis causes multiple organ system failure as a result of an overproduction of severe inflammatory responses after an inflammatory insult (*e.g.*, heart, lung, brain, liver, kidney, pancreas), and there is usually also failure of other organs involved in the response^2,3^. The lung and kidney generally experience early injury during the sepsis cascade process^3^. Clinically, patients can exhibit a combination of symptoms such as confusion, fever (also known as pyrexia), low blood pressure (hypotension), and/or decreased urine volume (oliguria). If not treated rapidly, these symptoms can develop into catastrophic complications including but not limited to coagulopathy, acute respiratory distress syndrome, and acute kidney injury^4,5^.

The pathophysiology of sepsis depends on an altered permeability of endothelial cells. Macrophages and neutrophils are activated and recruited to sites of infection by the action of bacterial endotoxins. Once there, these immune cells produce an overwhelming amount of pro-inflammatory cytokines (chemokines) and nitrogen oxides (NO), which leads to vasodilation or vascular hypotension due to the loss of vascular tone^6^. Also, when blood vessels become leaky (increased blood vessel permeability), it creates small blood clots (microthrombi), which reduces blood flow to the tissues, allowing for accumulations of reactive oxygen species and ultimately leading to organ failure at the cellular level^7,8^.

Nicotinamide mononucleotide (NMN) has gained importance as a molecule that plays a crucial role as a nucleotide and serves as an important precursor to nicotinamide adenine dinucleotide (NAD+)^9^. Playing a vital role in cellular energy metabolism, NMN restores NAD+ levels by acting as a substrate for mitochondrial enzymes^10^. It has been reported that NMN can normalize the NAD+/NADH ratio, which has a protective effect against oxidative stress^11^. Nicotinamide treatment has been reported to improve mortality and provide protection against acute liver injury in endotoxemia and perforation-induced sepsis models^12^. Nicotinamide riboside (NR), found in milk-based products, is metabolized to NMN, a precursor of NAD, via the enzymes NRK1 and NRK2^13^.

In the literature, it has been reported that NMN reduces lipopolysaccharide (LPS)-induced inflammation in macrophages^14^ and improves contractile function in mice^15^. NMN restored cardiac NAD levels in an ischemia-reperfusion model^16^ and improved metabolic balance in mice with type 2 diabetes^17^. It has also been found to increase NAD content and survival rate in rat hemorrhagic shock models^18^ and reduce LPS-induced lung^19^ and kidney damage^20^. In sepsis models induced by cecal ligation and puncture, NMN has been reported to reduce mortality by regulating the inflammatory response^21^.

The aim of this study was to investigate the potential protective effects of NMN administration on kidney tissue in experimental sepsis induced by cecal ligation and puncture (CLP) using histopathological methods.

## Methods

This study was approved by the Burdur Mehmet Akif Ersoy University Local Animal Experiments Ethics Committee, with decision number 1,320 dated on May 6, 2024. In the study, 28 female Sprague Dawley rats (8 weeks old, 200–250 g) obtained from the Burdur Mehmet Akif Ersoy University Experimental Research Center were used. The animals were kept in standard cages at 22 ± 2°C, with 50–60% humidity and a 12-hour light/dark cycle. *Ad libitum* (free) access to water and standard feed were provided throughout the experiment. All experimental procedures were performed in accordance with the National Institutes of Health Guidelines for the Care and Use of Laboratory Animals.

The rats were randomly divided into four equal groups (n = 7):

- Control group: healthy group with no procedures performed;
- Sham group: group that underwent only laparotomy, but without CLP;
- Sepsis (CLP) group: group in which sepsis was induced by cecal CLP;
- CLP + NMN group: group in which NMN (500 mg/kg, intraperitoneal) was administered 30 minutes before and 12 hours after the CLP procedure^22^.

### Sepsis model: cecal ligation and puncture procedure

The sepsis model was created using the CLP technique described in the literature^23^. Rats were anesthetized intraperitoneally (i.p.) with a combination of ketamine (80 mg/kg) and xylazine (10 mg/kg). After preparing the abdominal region under aseptic conditions, a 2-cm midline incision was made. The cecum was exposed and ligated with 4-silk sutures in the proximal 1/3 portion. The ligated cecum was punctured three times with an 18 G needle and gently squeezed to allow fecal contents to seep into the peritoneum. After the cecum was returned to its anatomical position, the laparotomy layers were closed with 4-sterile-silk sutures. The sham group underwent only laparotomy; the cecum was not ligated or punctured. All surgical procedures were performed by the same researcher to prevent experimental variability.

Twenty-four hours after the CLP procedure considering the peak period of cytokine storm and organ damage reported in the literature, all rats were painlessly euthanized by a high dose of thiopental sodium (0.5 g/kg, i.p.)^24,25^. Following euthanasia, decapitation was performed, and kidney tissues were rapidly excised.

### Tissue processing

Kidney tissue samples were fixed in 10% neutral buffered formalin for 48 hours, gradually dehydrated to remove water content using graded concentrations of ethanol (from 50 to 70%, 96%, and finally absolute alcohol)^26^ and cleared with xylene to improve transparency for paraffin infiltration^27^. The tissues were then embedded into paraffin wax to form blocks that offered support for accurate sectioning^28^. Sections were cut from the paraffin blocks with a rotary microtome (Leica RM2125) at a thickness of 4 µm. Sections were placed on microscope slides, ensuring smooth and wrinkle-free adhesion.

### Hematoxylin and eosin staining

The slides were placed in an oven at 60°C for 30 minutes and then went through deparaffinization, which was carried out by immersing the slides three times for 5 minutes each in xylene. This was followed by gradual rehydration of the sections through a series of alcohols consisting of 100% ethanol for 5 minutes; 96% ethanol for 3 minutes; 70% ethanol for 3 minutes; and finally distilled water for 2 minutes, which prepared the tissue for further staining. To stain acidic structures such as nucleic acid cytoplasmic components were immersed for 5 minutes in Mayer’s hematoxylin solution, which stains those structures blue or purple. After hematoxylin staining, the sections were rinsed off in running tap water for 1 minute to wash away the excess hue. The sections were differentiated with acid-alcohol (1% hydrochloric acid in 70% ethanol) for 1–2 seconds and rinsed off again in running water for 1 minute to remove the excess acid-alcohol mixture used for differentiation. Thereafter, sections were counter-stained with eosin Y solution for 1 minute, which colored the cytoplasm, and rinsed quickly with distilled water to remove additional staining solution while preventing much eosin from being lost^29,30^. The sections were dehydrated through a series of graded alcohols: 70% ethanol (1 min), 96% ethanol (1 min), 100% ethanol (2 exchanges, 2 minutes each). The sections were then cleared in xylene by immersing them three times for 5 minutes each to ensure adequate tissue transparency for mounting^31^. Finally, dehydrated and cleared sections were mounted with Entellan and permitted to dry at room temperature. The stained sections were evaluated under a light microscope (Nikon Eclipse i50, Tokyo, Japan) with various magnifications to check for tissue morphology^31^.

### Semi-quantitative histopathological scoring

To provide an objective assessment of renal histopathological alterations, a semi-quantitative scoring system was used. Histopathological evaluation was performed independently by two blinded histologists using 10 randomly selected non-overlapping fields from each kidney section at 200× magnification. Tubular injury was graded on a scale of 0–4 according to the percentage of affected tubules (0 = normal histology, 1 = < 25% involvement, 2 = 25–50% involvement, 3 = 50–75% involvement, and 4 = > 75% involvement). Glomerular congestion and inflammatory cell infiltration were graded on a scale of 0–3 according to severity (0 = absent, 1 = mild, 2 = moderate, and 3 = severe). For each animal, the mean score obtained from all evaluated fields was calculated and used for statistical analysis.

### Statistical analysis

Semi-quantitative histopathological scores are presented as mean ± standard deviation (SD). The normality of data distribution was assessed using the Shapiro–Wilk’s test. Differences in tubular injury, glomerular congestion, and inflammatory cell infiltration scores among groups were analyzed using one-way analysis of variance (ANOVA) followed by Tukey’s multiple comparison test. Statistical analyses were performed using GraphPad Prism 10 (GraphPad Software, San Diego, CA, United States of America). A *p*-value < 0.05 was considered statistically significant.

## Results

### Histopathological results

Control group glomeruli and proximal and distal tubules maintained their normal histological architecture. Glomeruli exhibited normal morphology with well-preserved capillary structures and a Bowman’s space that was within normal anatomical boundaries. Tubular epithelial cells were arranged regularly, with prominent, well-positioned nuclei. Structures of Henle’s loops and collecting ducts of medulla maintained normal histological features with normal cellular orientation.

Likewise, the sham group showed cortical structures maintained like the control group. No marked change was noted in either glomerular or tubular morphology. Medullary structures of Henle’s loops and collecting ducts remained intact without any signs of inflammation or cellular injury.

In the sepsis group, prominent structural alterations were noted in the glomeruli, such as glomerular congestion and infiltration of inflammatory cells. Most glomeruli showed fibrotic changes with a significant compromise of their structural integrity. Some atrophic glomeruli were identified, and coagulation was observed within the glomerular capillaries (shown in the inset picture). Bowman’s space was significantly dilated in most renal corpuscles. The proximal tubule diameter was reduced. Hypertrophy or degenerative changes with eosinophilic cytoplasm and pyknotic nuclei were seen in some proximal and distal tubular cells and juxtaglomerular cells were noted. Infiltration of inflammatory cells was detected throughout both cortical and medullary areas. Interstitial tissue showed fibrotic remodeling and infiltration of inflammatory cells. There were signs of mild vascular congestion and alteration of vascular wall structures. On the medulla, Henle’s loop and collecting ducts underwent degenerative changes, disorganization of cells, and marked infiltration of interstitial inflammatory cells.

**Figure 1:**
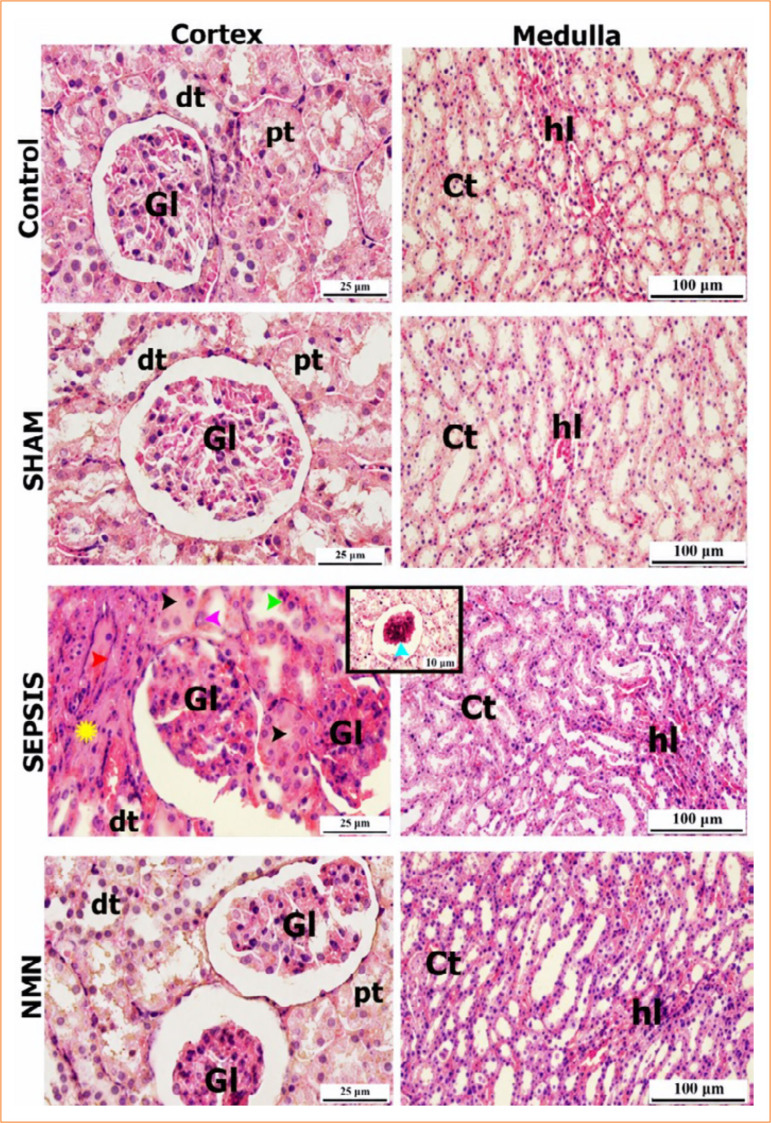
Representative hematoxylin and eosin-stained kidney sections from control, sham, sepsis and sepsis + nicotinamide mononucleotide groups (100× and 400×).

In the NMN group, the general glomerular architecture was better preserved, with far less congestion and inflammatory cell infiltration compared to the sepsis group. Fewer atrophic glomeruli were observed, and the extent of Bowman’s space dilation was significantly decreased as compared to sepsis group. Overall, the integrity of tubular epithelial structures was mostly maintained, with minimal degenerative changes. In the medulla, Henle’s loops and collecting ducts maintained normal morphological features. However, inflammatory cell infiltration was found surrounding some Henle’s loops, while localized fibrotic changes were noted in the interstitial connective tissue. Cellular organization in the collecting ducts was mostly preserved, while focal luminal dilation was noted (Fig. 1).

Gl: glomerulus; pt: proximal tubule; dt: distal tubule; hl: loops of Henle; Ct: collecting tubules; blue arrowhead: glomerular atrophy and thrombus formation; green arrowhead: cells with eosinophilic cytoplasm and pyknotic nuclei; pink arrowhead: hypertrophic cells; red arrowhead: impaired vascular wall; yellow star: fibrotic interstitial connective tissue. Source: Elaborated by the authors.

### Semi-quantitative histopathological results

**Figure 2:**
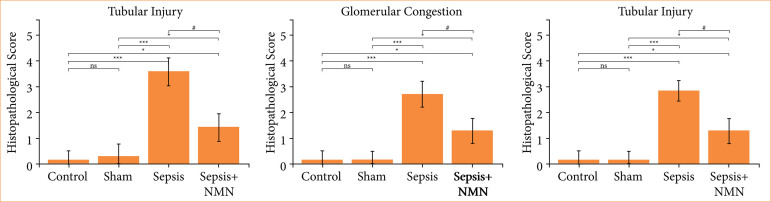
Semi-quantitative histopathological scoring of renal injury. Tubular injury score, glomerular congestion score, and inflammatory cell infiltration score in the control, sham, sepsis, and sepsis + nicotinamide mononucleotide groups. Data are presented as mean ± standard deviation (n = 7 animals per group). Statistical significance was determined using one-way analysis of variance followed by Tukey’s multiple comparison test.

Semi-quantitative histopathological scoring corroborated the qualitative microscopic findings. Tubular injury, glomerular congestion, and inflammatory cell infiltration scores were significantly higher in the sepsis group than in the control and sham groups (*p* < 0.001). Compared with the sepsis group, the sepsis + NMN group exhibited significantly lower tubular injury, glomerular congestion, and inflammatory cell infiltration scores (*p* < 0.05). No significant differences were observed between the control and sham groups (Fig. 2).

ns: not significant; *p < 0.05 versus the control and sham groups; ***p < 0.001 versus the control and sham groups; #*p* < 0.05 versus the sepsis group. Source: Elaborated by the authors.

## Discussion

In this study, the protective effects of NMN on sepsis-induced acute kidney injury (AKI) were investigated at the histopathological level using a CLP model. The data obtained revealed that severe glomerular and tubular injury occurring in the sepsis group was significantly alleviated with NMN treatment, and tissue integrity was largely preserved.

Sepsis is a complex clinical condition leading to multiple organ failure because of the uncontrolled inflammatory response of the immune system to infection^32^. AKI is one of the most common complications of sepsis and directly affects mortality. In the pathogenesis of sepsis-related AKI, pro-inflammatory cytokine storm, oxidative stress, endothelial dysfunction, and mitochondrial damage play a key role^33,34^. These processes lead to a decrease in renal blood flow, tubulointerstitial fibrotic changes and consequently dramatic decreases in glomerular filtration rate.

The Bowman’s capsule dilation, tubular cell degeneration, and intense interstitial inflammatory infiltration observed in the sepsis group in our study parallel the findings of renal perfusion impairment and peritubular capillary damage reported by Seely et al.^35^.

NMN is a critical nucleotide that restores cellular metabolism and energy balance by increasing intracellular NAD+ levels. In the literature, it is known that a decrease in NAD+ levels is directly related to mitochondrial dysfunction, cellular senescence, and increased oxidative stress^36^. It is suggested that NMN application reduces cellular stress by supporting the NAD+ pool and has a cytoprotective effect in organs with high energy demand, such as the kidney^16,37^. The reduction in glomerular congestion and the preservation of tubular epithelial structure observed in the NMN group in our study support this metabolic supportive effect of NMN on renal parenchyma.

Our current findings are consistent with previous research^14,38^ showing that NMN suppresses macrophage-derived inflammation and slows the fibrotic process. Zhou et al.^39^ highlighted that NAD+ increase improves mitochondrial damage in systemic inflammation and heart failure. Similarly, in our study, the reduction in inflammatory cell infiltration observed following NMN treatment suggested that this molecule may modulate the detrimental effects of systemic inflammation on renal tissue.

The importance of oxidative damage in sepsis pathogenesis has also been highlighted in studies in which different treatment agents have been tested. For example, Çavuşoğlu et al.^40^ reported that oxytocin improved oxidative stress markers in sepsis-induced renal damage. Guo et al.^41^ reported the same for ginsenoside Rg1. The decrease in the number of pyknotic nuclei and the minimal level of eosinophilic cytoplasmic changes (degeneration) that we observed in the NMN group provided morphological evidence suggesting that NMN may protect tissue architecture. Su et al.^42^, while discussing the relationship between cellular stress and inflammation in the CLP model, stated that anti-inflammatory strategies are critical for kidney health.

In our study, the fact that NMN normalized the widening of the Bowman’s space, which was particularly evident in the sepsis group, and reduced the number of atrophic glomeruli suggested that renal microcirculation may have been preserved. The high bioavailability and low toxicity profile of NMN make this molecule a promising candidate for sepsis management. A recent study showed that NMN supplementation alleviated sepsis-related acute kidney injury by activating the NAD+/SIRT3 signaling pathway^43^. This finding was consistent with our observations in the NMN group, in which tubular epithelial integrity was preserved and cellular degeneration remained minimal. In the literature, NMN has been reported to reduce pro-inflammatory cytokine release and immune cell infiltration by inhibiting P38 and P65 (NF-κB) phosphorylation in sepsis models^44^.

The significant reduction in inflammatory cell infiltration in both cortical and medullary regions of rats treated with NMN in our study can be explained by the potential inhibitory effect of NMN on these molecular signaling pathways. It has been reported that NMN can inhibit DNA damage and cellular senescence in tubular cells^45^. This information is a critical mechanism supporting the near normalization of pyknotic nuclei and eosinophilic cytoplasmic changes that we detected in the sepsis group with NMN treatment. Semi-quantitative histopathological scoring provided objective support for the microscopic observations. Consistent with the qualitative findings, sepsis induced marked tubular injury, glomerular congestion, and inflammatory cell infiltration, whereas NMN administration significantly reduced all evaluated histopathological injury parameters. These findings further support the renoprotective effect of NMN in CLP-induced sepsis.

A limitation of the present study was the lack of biochemical renal function parameters, such as serum creatinine and blood urea nitrogen levels. Consequently, the histopathological findings could not be directly correlated with renal functional outcomes. Future studies incorporating histopathological, biochemical, and molecular assessments are warranted to provide a more comprehensive evaluation of the renoprotective effects of NMN in sepsis-associated acute kidney injury.

## Conclusion

This study demonstrates that NMN protects kidney tissue architecture and significantly reduces morphological damage in an experimental sepsis model caused by CLP, as revealed by histopathological findings. The data obtained support the idea that NMN has cytoprotective and anti-inflammatory potential against sepsis-induced AKI and may be effective in maintaining glomerular and tubular integrity. NMN is a promising therapeutic agent candidate in the destructive process of sepsis leading to multiple organ failure. However, more comprehensive preclinical and clinical studies are needed to fully understand the clinical efficacy and renal protection mechanisms of NMN, which will examine different dose regimens, application durations, and advanced molecular signaling pathways.

## Data Availability

Upon request, researchers will be granted access to the dataset utilized in this study.
